# Retroperitoneal mucinous cystadenoma with neuroendocrine differentiation: a rare case and comprehensive approach to diagnosis and management

**DOI:** 10.1186/s13000-025-01658-7

**Published:** 2025-05-03

**Authors:** Yen Ho, Wei-Yu Chen, Chung-Howe Lai, Syuan-Hao Syu

**Affiliations:** 1 Department of Urology, Wan Fang Hospital, Taipei Medical University, Taipei, Taiwan; 2 Department of Pathology, Wan Fang Hospital, Taipei Medical University, Taipei, Taiwan; 3 Departments of Pathology, School of Medicine, College of Medicine, Taipei Medical University, Taipei, Taiwan; 4 Department of Urology, School of Medicine, College of Medicine, Taipei Medical University, Taipei, Taiwan; 5 Graduate Institute of Clinical Medicine, College of Medicine, Taipei Medical University, Taipei, Taiwan

**Keywords:** Retroperitoneal mucinous cystadenoma, Neuroendocrine differentiation, Surgical excision, Histopathological analysis

## Abstract

**Background:**

Retroperitoneal mucinous cystadenomas are exceptionally rare neoplasms, with limited cases reported in the literature. The occurrence of neuroendocrine differentiation in such tumors is even more uncommon, posing unique diagnostic and management challenges.

**Case presentation:**

We report a case of a 32-year-old woman who was incidentally diagnosed with a right retroperitoneal cyst during routine prenatal ultrasonography. The patient remained asymptomatic until postpartum, prompting further evaluation of the cyst. Imaging studies identified a large cystic mass, ultimately leading to diagnostic laparoscopy and surgical excision. Histopathological analysis confirmed the diagnosis of a mucinous cystadenoma with neuroendocrine cell proliferation.

**Discussion:**

This case highlights the complexity of diagnosing and managing retroperitoneal mucinous cystadenomas, particularly those with neuroendocrine features. Given the rarity of these tumors, thorough histopathological examination is crucial to differentiate them from other cystic lesions. Surgical excision remains the definitive treatment, with long-term follow-up essential to ensure complete resolution and monitor for recurrence or malignant transformation.

**Conclusion:**

Retroperitoneal mucinous cystadenomas with neuroendocrine differentiation represent a rare clinical entity requiring careful evaluation. This report underscores the importance of considering neuroendocrine differentiation in retroperitoneal cystic lesions and emphasizes the role of complete surgical excision followed by close monitoring to ensure favorable outcomes.

## Introduction

Retroperitoneal mucinous cystadenomas (RMCs) are exceedingly rare neoplasms, predominantly affecting women and typically detected incidentally due to their asymptomatic nature. While these tumors are well-documented in association with the ovaries or pancreas, their presence in the retroperitoneum is highly unusual, complicating both diagnosis and management strategies. Various hypotheses have been proposed regarding the origin of RMCs, including Müllerian duct remnants, ectopic ovarian tissue, or mucinous metaplasia of mesothelial cells. Recent studies have suggested that these cells could undergo metaplasia under certain hormonal influences or microenvironmental factors, contributing to cyst development [1–3].

The clinical diagnosis of RMCs remains challenging, as symptoms, when present, are often vague and nonspecific, such as mild abdominal discomfort or a palpable mass. Imaging modalities, particularly computed tomography (CT) and magnetic resonance imaging (MRI), are indispensable for detecting these cystic lesions. However, distinguishing RMCs from other retroperitoneal cystic neoplasms remains difficult, especially in differentiating between benign and malignant masses. Advanced imaging techniques, such as diffusion-weighted MRI and Positron Emission Tomography (PET), may offer better specificity and assist in the differential diagnosis of retroperitoneal masses [4, 5]. Neuroendocrine differentiation, which has been observed in rare cases, adds an additional layer of complexity that necessitates meticulous histopathological and immunohistochemical examination [6, 7].

Surgical resection is the treatment of choice for RMCs to prevent recurrence and minimize the risk of malignant transformation. Long-term follow-up is particularly critical in cases with neuroendocrine features, as such tumors may exhibit a more aggressive behavior and higher recurrence potential [3, 8].

## Case presentation

A 32-year-old woman with no significant medical or surgical history was incidentally found to have a right-sided abdominal cyst during routine prenatal ultrasonography approximately one year before her presentation. She remained asymptomatic throughout her pregnancy and the postpartum period, without any complaints of abdominal pain, vaginal discharge, gastrointestinal disturbances, or urinary symptoms.

Following an uncomplicated vaginal delivery, the patient sought further evaluation for the cyst. Approximately three and a half months later, laboratory testing revealed normal levels of tumor markers, including carbohydrate antigen-125 (CA-125), CA-153, and CA-199. Two months after these tests, transvaginal ultrasonography identified a 139 mm cystic mass in the right ovary.

**Fig. 1:**
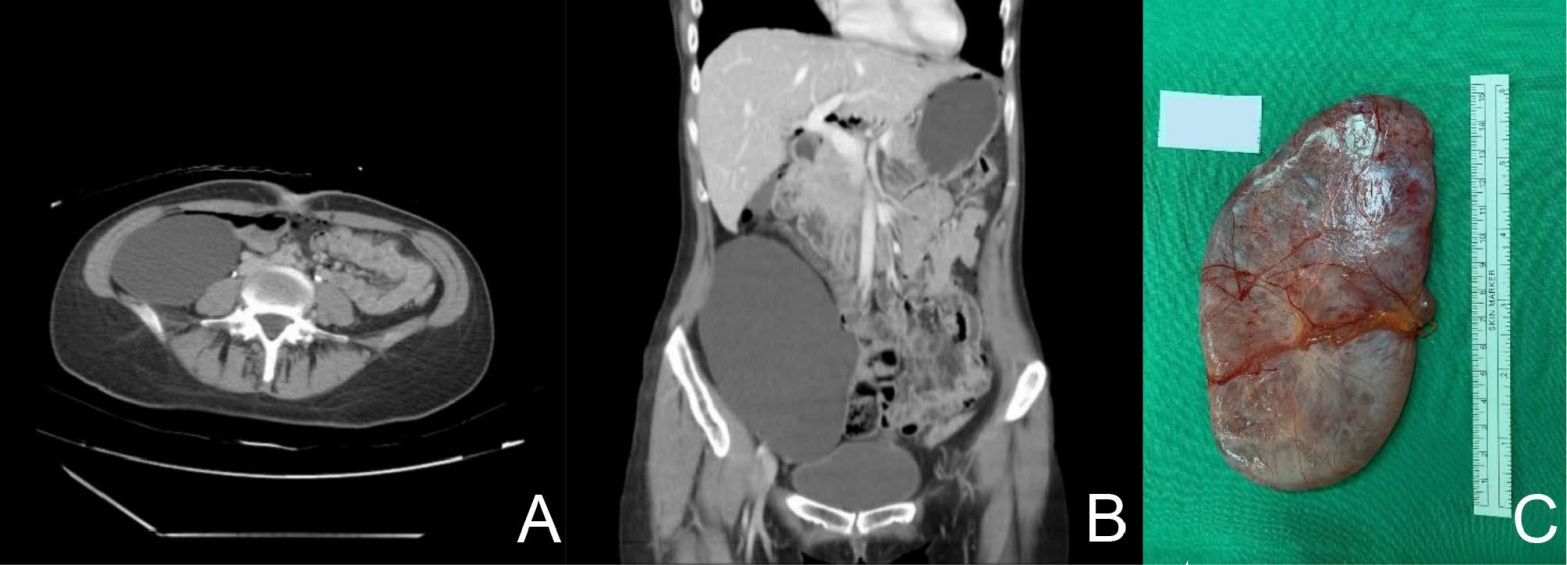
Radiologic and gross features of the retroperitoneal mucinous cystadenoma. **A**, **B**, Abdominal and pelvic CT scans showing a well-circumscribed retroperitoneal cystic lesion measuring 11 × 17.6 cm, with thin walls, no solid enhancing components, and no invasion of adjacent structures—features consistent with a benign lesion; **C**,Gross specimen after excision, revealing a unilocular cyst with a smooth external surface and a thickened base

A diagnostic laparoscopy performed shortly thereafter revealed a large retroperitoneal cyst measuring 14 × 9 cm, with no involvement of the bilateral adnexa. Abdominal and pelvic CT confirmed the presence of a well-defined retroperitoneal cystic lesion measuring 11 × 17.6 cm (Fig. 1A, B).

A few days later, the patient underwent surgical excision of the cyst by a urologic team. Intraoperatively, a cyst with a thickened base was isolated, and aspiration yielded 480 mL of clear fluid, which subsequently gelatinized. The postoperative course was uneventful, and the patient was discharged two days after surgery without complications.

Three months after the surgery, a follow-up CT of the abdomen and pelvis revealed no recurrence of the lesion, with minimal residual retroperitoneal air in the right presacral region, indicating complete resolution. Subsequent imaging studies over the next three years confirmed the absence of recurrence, demonstrating a successful and lasting outcome.

The resected specimen consisted of a single, well-circumscribed tissue fragment measuring 10.5 × 9.9 × 5.5 cm (Fig. 1C). On gross examination, the external surface appeared soft with a gray-to-reddish coloration. Cytologic evaluation of the aspirated fluid, which had gelatinized after collection, was also performed.

**Fig. 2:**
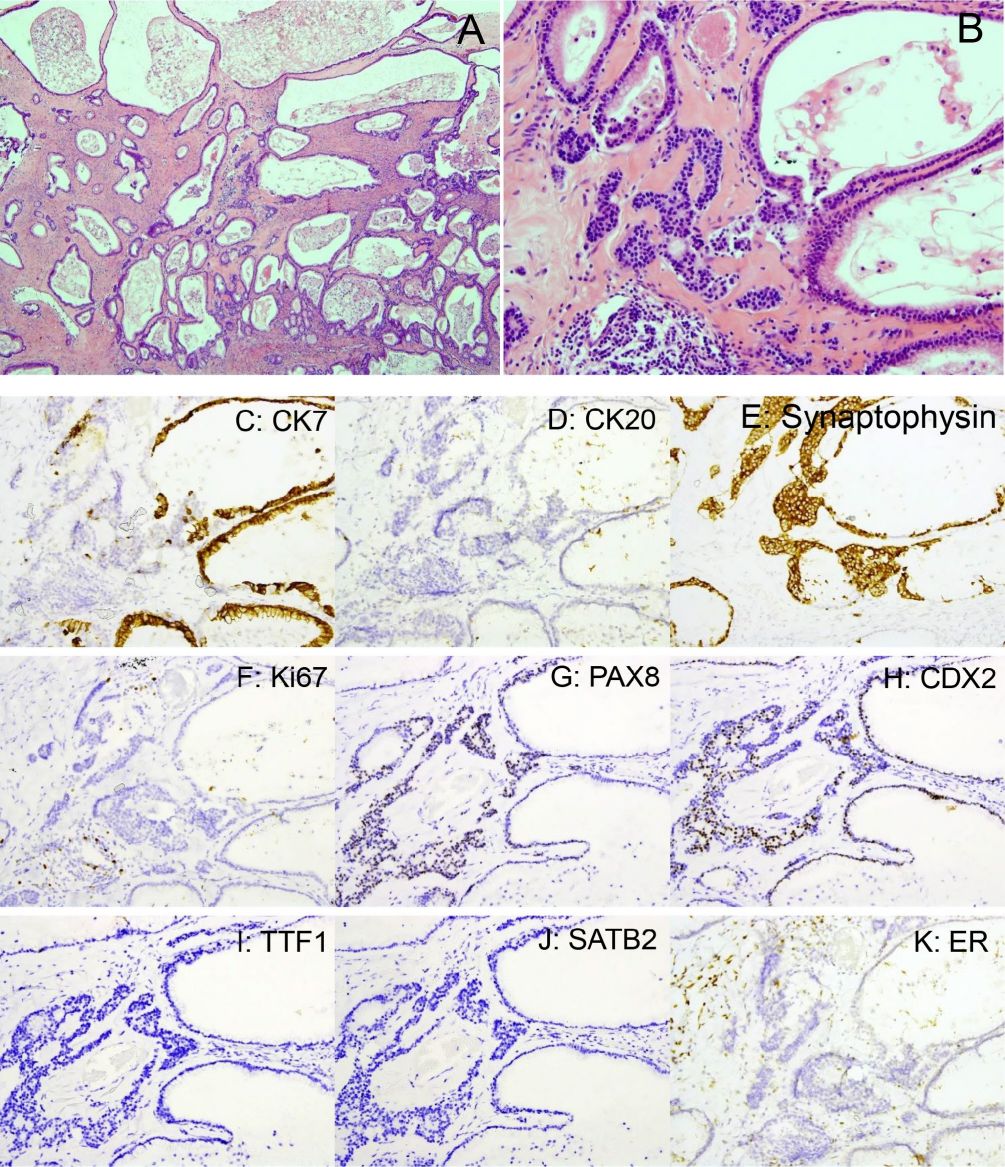
, Histopathological and immunohistochemical features of mucinous cystadenoma with neuroendocrine differentiation. **A**, Low-power view (H&E, 40×) of the mucinous cystadenoma; **B**, High-power view (H&E, 200×) demonstrating monotonous small neuroendocrine cells arranged in solid nests within the stromal component; **C**, CK7 immunostaining highlights the mucinous epithelium (positive), with negative staining in neuroendocrine cells; **D**, CK20 is negative in both mucinous and neuroendocrine components; **E**, Synaptophysin is strongly positive in neuroendocrine cells but negative in mucinous epithelium; **F**, Ki-67 shows a very low proliferative index (< 2%) in neuroendocrine cells; **G**, PAX8 is focally positive in both mucinous epithelium and neuroendocrine cells, **H**, CDX2 shows focal positivity in both components; **I**, TTF-1 is negative in both mucinous and neuroendocrine cells; **J**, SATB2 is negative in both components, **K**, Estrogen receptor is negative in both epithelial components but positive in the stromal (ovarian-type) cells

Microscopic examination revealed that the cyst was predominantly lined by mucinous epithelium, composed of simple, flat, or columnar epithelial cells without nuclear atypia, consistent with a mucinous cystadenoma (Fig. 2A). In some areas, atypical proliferation of the mucinous epithelium with gland budding and mild to moderate nuclear atypia was observed, involving less than 10% of the tumor volume (Fig. 2B). Immunohistochemically, the mucinous epithelium was positive for cytokeratin-7 (CK7) and focally positive for Caudal-type homeobox 2 (CDX2) and Paired box 8 (PAX8), while negative for CK20, estrogen receptor (ER), Thyroid transcription factor 1 (TTF-1), and Special AT-rich sequence-binding protein 2 (SATB2) (Fig. 2C–K). The stromal cells showed positivity for ER, consistent with ovarian-type stroma.

Notably, neuroendocrine cell proliferation was identified, characterized by monotonous small cells with scant cytoplasm, arranged in solid nests or admixed with mucinous epithelium in the stroma (Fig. 2B). These cells were strongly positive for synaptophysin and chromogranin A, confirming neuroendocrine differentiation. They were negative for CK7, CK20, ER, TTF-1, and SATB2, but showed focal positivity for CDX2 and PAX8 (Fig. 2C–K). The Antigen identified by monoclonal antibody Ki-67 (Ki-67) proliferative index in the neuroendocrine component was low (< 2%), indicating a low proliferative activity (Fig. 2F).

Cytological analysis of the aspirated fluid revealed the presence of scattered histiocytes, lymphocytes, and neutrophils, indicative of a mild localized inflammatory response. No evidence of malignancy was detected in the specimen. The findings were consistent with a benign retroperitoneal mucinous cystadenoma with areas of neuroendocrine differentiation.

## Discussion

Retroperitoneal mucinous cystadenomas represent an uncommon and poorly understood subset of cystic neoplasms. Our case aligns with the histopathological spectrum outlined in the largest reported series by Roma and Malpica, which classified PRMCs into mucinous cystadenomas, tumors of low malignant potential, and mucinous carcinomas based on architectural complexity and cytologic atypia [3]. Their histogenesis is still a matter of debate, with hypotheses ranging from Müllerian duct remnants and ectopic ovarian tissue to mucinous metaplasia of mesothelial cells. This case illustrates the challenges associated with diagnosing these tumors, particularly given their unusual location in the retroperitoneum. The embryological origin of RMCs, though controversial, is crucial to understanding the tumor’s characteristics and guiding treatment decisions [2, 4, 5].

The presence of neuroendocrine differentiation in RMCs adds significant complexity to both diagnosis and prognosis. Neuroendocrine tumors (NETs) typically arise from organs with a natural presence of neuroendocrine cells, such as the pancreas, adrenal glands, and gastrointestinal tract. The coexistence of neuroendocrine differentiation within RMCs is extremely rare and suggests potential mechanisms of cellular metaplasia or dedifferentiation, possibly influenced by local microenvironmental factors or genetic mutations [7, 9]. Immunohistochemical markers, such as synaptophysin and chromogranin, are instrumental in confirming neuroendocrine differentiation, and studies have suggested that the presence of these markers may correlate with a more aggressive clinical course and higher risk of recurrence [10, 11]. In our case, the mucinous epithelium was positive for CK7 and focally positive for CDX2 and PAX8, while negative for SATB2, ER, and TTF-1. The neuroendocrine component showed strong expression of synaptophysin and chromogranin A, confirming its identity, and was also focally positive for CDX2 and PAX8. These findings suggest a degree of gastrointestinal-like and possible Müllerian differentiation, although the full histogenetic origin remains unclear. This immunoprofile supports the hypothesis that these tumors may arise from ectopic Müllerian epithelium or multipotent progenitor cells in the retroperitoneum. In their series of 18 cases, Roma and Malpica reported that primary retroperitoneal mucinous tumors frequently exhibited diffuse CK7 and focal CK20 positivity, a pattern also seen in ovarian mucinous tumors. These findings support a possible shared histogenetic origin, particularly in cases with ovarian-type stroma [3].

Although recent reports have contributed to our clinical and radiological understanding of primary RMCs [12–14], few have addressed their molecular or genetic background. Recent studies, however, have identified key genetic alterations implicated in the pathogenesis of mucinous cystic neoplasms and neuroendocrine tumors. Kirsten rat sarcoma viral oncogene homolog (KRAS) and GNAS mutations are frequently found in mucinous neoplasms of the pancreas and appendix, suggesting a role in tumor initiation and progression [15]. Tumor protein p53 (TP53) mutations are more commonly associated with high-grade or malignant transformation. In neuroendocrine tumors, mutations in Multiple endocrine neoplasia type 1 (MEN1), Death-domain associated protein (DAXX), and Alpha-thalassemia/mental retardation syndrome X-linked (ATRX), along with alterations in the Mammalian target of rapamycin (mTOR) pathway, have been linked to tumor biology and therapeutic response [16]. Notably, mutations in MEN1/DAXX/ATRX are associated with improved progression-free survival in gastroenteropancreatic NETs treated with peptide receptor radionuclide therapy, suggesting potential clinical relevance as predictive biomarkers [17]. Although these molecular profiles are primarily derived from gastrointestinal and pancreatic cases, they may provide insight into the genetic underpinnings of retroperitoneal mucinous neoplasms with neuroendocrine differentiation. Further molecular characterization in such rare cases is warranted.

The implications of neuroendocrine differentiation on treatment and prognosis are significant, as tumors with neuroendocrine components may behave more aggressively than those without such features. For this reason, it is recommended that patients with neuroendocrine-differentiated RMCs undergo more rigorous follow-up protocols, including frequent imaging and tumor marker evaluation to identify recurrence or malignant progression at an early stage [8, 18].

Diagnosing retroperitoneal mucinous cystadenomas is inherently difficult due to their nonspecific clinical presentation. Many cases, as demonstrated in this study, are diagnosed incidentally during imaging conducted for unrelated reasons. In our case, abdominal and pelvic CT revealed a well-circumscribed retroperitoneal cystic lesion measuring 11 × 17.6 cm, with thin walls, no enhancing solid components, and no evidence of invasion into adjacent organs. These imaging characteristics strongly suggested a benign etiology and were essential in guiding the decision to proceed with laparoscopic surgical excision. While CT and MRI remain critical tools for evaluating retroperitoneal cystic lesions, the specific radiologic features in this case—particularly the absence of solid nodules or infiltrative margins—played a key role in differentiating the lesion from potentially malignant masses [5, 7, 19]. Recent advancements, such as diffusion-weighted MRI and PET, have shown promise in improving diagnostic specificity, particularly in distinguishing between benign and malignant lesions based on tissue composition and metabolic activity [4, 6].

Tumor markers, such as CA-125 and CA19-9, have demonstrated some utility in differentiating benign from malignant lesions. However, their sensitivity and specificity are limited, and their role should be considered adjunctive rather than definitive. New biomarkers and molecular techniques are emerging as potential tools for more accurate diagnosis [8, 15, 20].

Surgical excision remains the cornerstone of treatment for retroperitoneal mucinous cystadenomas, with the primary goal being to ensure complete removal of the tumor to prevent recurrence and minimize the risk of malignant transformation. Open and laparoscopic approaches are both viable options, with the choice often depending on the size and location of the tumor, as well as the surgeon’s expertise. Complete resection is associated with a favorable prognosis, as observed in the present case, where successful removal led to an absence of recurrence during the initial follow-up period [5, 9].

However, in cases where neuroendocrine differentiation is present, the prognosis is more guarded. These patients may require more intensive postoperative follow-up, including regular imaging and biomarker evaluations. Advances in surgical techniques, including minimally invasive approaches, may help reduce postoperative morbidity while ensuring complete tumor removal [9, 18]. Additionally, the role of preoperative biopsy in tailoring the surgical approach remains an important consideration for achieving complete resection without complications [19, 20].

While several recent case reports have documented retroperitoneal mucinous cystadenomas [12–14], none have described associated neuroendocrine differentiation or performed a comprehensive immunohistochemical characterization. The presence of a confirmed neuroendocrine component in our case—supported by synaptophysin, chromogranin A, CDX2, and Ki-67 profiling—provides new insight into the potential histologic and phenotypic diversity of these rare neoplasms.

Given the rarity of retroperitoneal mucinous cystadenomas, particularly those with neuroendocrine differentiation, each reported case contributes valuable insights into the biological behavior of these tumors and their optimal management. While current treatment strategies are largely extrapolated from ovarian mucinous cystadenomas, further research is needed to establish specific guidelines tailored to retroperitoneal variants. Future studies should focus on the molecular and genetic characteristics of these tumors, as this could lead to the development of targeted therapies [11, 20].

Long-term, multicenter studies are required to better understand the recurrence rates, prognostic factors, and treatment outcomes for patients with these rare neoplasms. Moreover, international collaboration and the establishment of a comprehensive database for retroperitoneal tumors could facilitate data collection and analysis, leading to the development of consensus guidelines for diagnosis, treatment, and follow-up of these challenging cases [9, 19].

## Conclusion

Retroperitoneal mucinous cystadenomas with neuroendocrine differentiation are extremely rare neoplasms, posing unique diagnostic and management challenges. This case report highlights the importance of maintaining a high index of suspicion for retroperitoneal cystic lesions, especially when neuroendocrine features are present. Surgical resection is essential for both diagnosis and definitive treatment, and thorough histopathological evaluation is critical to differentiate these lesions from other cystic neoplasms. The successful surgical outcome and absence of recurrence over a three-year follow-up period demonstrate that complete excision remains the best approach for managing these rare tumors. Continued reporting of similar cases is necessary to enhance our understanding and guide future treatment and follow-up protocols, ultimately contributing to the broader knowledge base on retroperitoneal cystic tumors.

## Data Availability

No datasets were generated or analysed during the current study.
